# The Multifaceted Role of Commensal Microbiota in Homeostasis and Gastrointestinal Diseases

**DOI:** 10.1155/2015/321241

**Published:** 2015-02-22

**Authors:** Marcelo José Barbosa Silva, Matheus Batista Heitor Carneiro, Brunna dos Anjos Pultz, Danielle Pereira Silva, Mateus Eustáquio de Moura Lopes, Liliane Martins dos Santos

**Affiliations:** ^1^Laboratório de Biomarcadores Tumorais e Osteoimunologia, Instituto de Ciências Biomédicas, Universidade de Uberlândia, Avenida Pará 1720, 38400-902 Uberlândia, MG, Brazil; ^2^Laboratório de Gnotobiologia e Imunologia, Departamento de Bioquímica e Imunologia, Instituto de Ciências Biológicas, Universidade Federal de Minas Gerais, ICB/UFMG, Avenida Antonio Carlos 6627, 31270-901, Belo Horizonte, MG, Brazil

## Abstract

The gastrointestinal tract houses a complex and diverse community of microbes. In recent years, an increased understanding of the importance of intestinal microbiota for human physiology has been gained. In the steady state, commensal microorganisms have a symbiotic relationship with the host and possess critical and distinct functions, including directly influencing immunity. This means that recognition of commensal antigens is necessary for the development of complete immune responses. Therefore, the immune system must face the challenge of maintaining mucosal homeostasis while dealing with undue passage of commensal or pathogenic microbes, as well as the host nutritional status or drug use. Disruption of this fine balance has been associated with the development of several intestinal inflammatory diseases. In this review, we discuss the mechanisms involved in the modulation of host-microbe interactions and how the breakdown of this homeostatic association can lead to intestinal inflammation and pathology.

## 1. The Normal Microbiota

It has been estimated that trillions of microbes inhabit our gastrointestinal tract (GIT), most of which reside in the distal intestine, where they synthesize essential vitamins and process indigestible components of our diet, such as plant polysaccharides. Furthermore, these microbes influence both normal physiology and disease susceptibilities [1].

The first step towards understanding the relationship between the host and microbes is the characterization of the normal microbiota and the differences that are associated with disease. Moreover, it has been reported that age, genetics, environment, and diet can alter the relationship of intestinal microbiota and host [2].

Eckburg and colleagues [3] showed that in adults most of the intestinal bacteria belong to just a few phyla. Bacteroidetes and Firmicutes are usually dominant, which is consistent with recent studies [4, 5]. Actinobacteria, Proteobacteria, Fusobacteria, and Verrucomicrobia phyla are frequent but generally minor constituents [3–5]. Our microbiota also contains methanogenic archaea (mainly* Methanobrevibacter smithii*), eukarya (mainly yeasts), and viruses [6].

In recent years, our knowledge regarding species and functional composition of the human intestinal microbiome has increased rapidly, but very little is known about the composition of this microbiome around the world. Arumugam and colleagues [7] characterized variations in the composition of the intestinal microbiota in 39 individuals from four continents by analyzing the fecal metagenome. The phylogenetic composition showed that the Firmicutes and Bacteroidetes phyla constitute the majority of the human intestinal microbiota. The* Bacteroides* genus was the most abundant but also the most variable among individuals. According to the variation between the microbiota, it was proposed that the intestinal microbial community could be stratified into three groups, called enterotypes. Each of these three enterotypes is identifiable by the variation in the levels of one of three genera:* Bacteroides* (enterotype 1),* Prevotella* (enterotype 2), and* Ruminococcus* (enterotype 3). Despite the stability of these three major groups, their relative proportions and the species present are highly variable between individuals.

Regarding bacterial stability another study analysis of fecal samples from 37 healthy adults showed that individual microbiota was notably stable over five years. Extrapolation of these data suggests that most of the bacteria present in the intestine were residents for decades. Bacteroidetes and Actinobacteria are significantly more stable than the average population [8]. Concerning the stability of Bacteroidetes, it was shown that these bacteria have evolved in species-specific physical interactions with the host that mediates stability, and the genetic locus commensal colonization factors (CFC) represents a novel molecular mechanism for symbiosis [9]. It is important to point out that the fecal microbiota differs from mucosal microbiota [3, 10]. Therefore, Siezen and Kleerebezem proposed a new term called “faecotypes” instead of “enterotypes,” since it is known that the microbial abundance and composition changes dramatically throughout the GIT, and perhaps “enterotypes” may not reflect the microbial composition of the whole intestine [11].

Although the intestinal microbiota is stable in adulthood, it undergoes fluctuations during childhood and old age. In children, the type of bacteria colonizing the intestine is defined very early according to the type of childbirth. Normal delivery is an important source of intestinal Actinobacteria, especially* Bifidobacterium*, while cesarean delivery provides a bacterial community similar to that found on the skin surface, dominated by* Staphylococcus* and the colonization by* Lactobacillus*,* Bifidobacterium*, and* Bacteroides* [12, 13]. In elderly individuals, there is a decreasing quantity and diversity of species of* Bacteroides* and* Bifidobacterium* and an increase in facultative anaerobe bacteria such as* Fusobacterium*,* Clostridium*, and* Eubacterium *species. Increase of these bacteria genus is harmful to host since they present high proteolytic activity, which is responsible for putrefaction of large bowel [14].

The majority of the gut microbes are harmless or beneficial to the host. However, studies of human microbiota composition have discovered that alterations in the microbiome composition are present in obese individuals [15], as well as in individuals with a variety of other diseases such, as inflammatory bowel diseases (IBDs) [16] and cancer [17]. Furthermore, antibiotic administration impacts the human intestinal microbiota. These antimicrobial agents contribute to the decrease of colonization resistance of members of the commensal microbiota, which can lead the development of a range of diseases, as well as the emergence antimicrobial resistance. Moreover, it was believed that the commensal microbiota could normalize a few weeks after treatment discontinuation, but this is not true for some specific members that may be affected for long periods of time [18].

## 2. Gut Microbiota, Nutrition, and Metabolism

The microbiome is strongly influenced by diet. This factor was suggested to be more of a determinant than hygiene, climate, ethnicity, and geography in a study comparing the gut microbial composition between children from a rural African village and a city in Europe [19]. Further, there was no difference in terms of the prevalence of the four major phyla found in the human gut (Actinobacteria, Bacteroidetes, Firmicutes, and Proteobacteria) when comparing a low-fat/high-fiber diet and a low-fiber/high-fat diet in different studies. However, there was a difference in terms of proportion between those phyla. More Actinobacteria and Bacteroidetes were found in low-fat/high-fiber diets, whereas more Firmicutes and Proteobacteria were found in low-fiber/high-fat diets [19, 20]. Another example of diet influencing human gut microbiota was shown by a study comparing populations in Russia and other countries. Russian subjects presented some specific populations of Firmicutes and Actinobacteria phyla, which were probably related to their diet, since those bacteria are specialized in starch metabolism, and starch-rich foods are typical in this country [21]. Furthermore, in a murine model, it was possible to relate specific components from the diet with the prevalence of different species of bacteria in the gut, which clearly shows the influence of diet in the composition of microbiota [22].

Diet administered to infants during the first six months of life is also important for the microbiota composition. Recent studies with infants in China showed different proportions of Actinobacteria and Bacteroidetes populations between breast-fed and formula-fed infants with a higher proportion of both types in the breast-fed diet [23]. Although the composition of microbiota is stable in healthy adults, diet can rapidly change the proportion of some bacterial populations in the gut, in less than 24 hours. Administration of a high-fat diet to humanized gnotobiotic mice increased the population of Firmicutes and decreased the Bacteroidetes population [24]. Interestingly, this change in human gut microbiota in response to an altered diet is faster in an animal-based diet than in a plant-based diet [25]. In addition, this effect varies for different populations of bacteria in the gut. Enterotypes are related to a long-term diet and thus were not affected in an experimental model until 10 days after the administration of a specific diet [20].

Evolution of the Western diet with the introduction of processed food and changes in nutritional characteristics of the human diet, especially in fiber, sugar, and fatty acid contents, have been proposed to be related to the increase of the incidence of chronic diseases [26, 27]. In this context, the composition of microbiota, which depends on the diet, is important because of the influence of bacteria metabolism for the production of important metabolites for the host [19, 24]. One relevant metabolite produced by fermentation of dietary fiber is the short-chain fatty acids (SCFAs). Acetate, butyrate, and propionate are the main SCFAs that result from fermentation of carbohydrates and amino acids in the diet [28]. The presence of these metabolites are microbiota-dependent, since rats and germ-free mice showed a small amount of SCFAs in the intestine, which was probably coming from the diet [29]. Short-chain fatty acids have been described as important anti-inflammatory molecules. Administration of acetate in drinking water was enough to decrease inflammation in a colitis experimental model. The mechanism seems to be through reduction of production of proinflammatory chemokines and cytokines, such as macrophage inflammatory protein 1-alpha (MIP-1*α*) and tumor necrosis factor alpha (TNF-*α*). In this way, mice treated with acetate showed less migration of neutrophils into the gut. Furthermore, this SCFA is important in reducing inflammation in other sites, and not only in the intestine. The effect of acetate through its binding to the G-protein-coupled receptor 43 (GPR43) is also relevant to control inflammation in experimental models of arthritis and asthma [30]. In addition, mice fed with a low-fiber diet showed higher cell infiltration in allergic airway inflammation. Furthermore, treatment of mice with propionate induced protective effects in this disease through G-protein-coupled receptor 41 (GPR41) and not the GPR43 receptor [31]. Interestingly, this study showed that dietary fiber can change the gut and lung microbiota, another consistent example of how diet can change the microbiome and how this can be important for the host [31]. These studies demonstrate how diets rich in fiber could attenuate proinflammatory diseases [30, 31].

The microbiota has been described as an important factor in modulation of host energy metabolism and even in the level of some lipid classes in the serum. The amounts of 18 phosphatidylcholine species and nine triglyceride species in serum of conventional mice were reduced compared with levels in germ-free mice [32]. Recent studies have associated normal microbiota with obesity. Interestingly, conventional mice showed a higher percentage of total body fat than germ-free mice, and conventionalization of those mice with fecal microbiota increased their body fat within only 10 days after their colonization. This effect cannot be associated with differences in metabolic rate or in chow consumed by those mice. The authors suggested that gut commensals may inhibit the expression of FIAF (fasting-induced adipose factor), which can block the production of LPL, an important lipase [33]. Also, the simple transplantation of microbiota from obese mice can induce weight gain in a murine model [34]. Furthermore, another study showed an interesting alteration in the composition of the main phyla of bacteria in the gut of* ob/ob* mice which are, by spontaneous mutation, deficient in leptin which leads to an increase in food intake and obesity phenotype [35]. A higher frequency of Firmicutes and a lower frequency of Bacteroidetes were found in these mice, which develop obesity [36]. The same pattern was also found in humans. Obese people were found to have more Firmicutes than Bacteroidetes but, after a diet therapy, they presented an increased amount of Bacteroidetes [37]. Composition of microbiota, in association with genotype and lifestyle, is an important factor in obesity. The microbiota from obese humans can even influence the production of some metabolites, which are typical of this disorder, including the general metabolism of amino acids [38].

## 3. Commensal Intestinal Bacteria and the Immune System

Although microbes are frequently seen as pathogenic, it is well established that most of them live in symbiosis with humans. Most of the microbes that inhabit the human intestine have a highly coevolved relationship with the immune system, which leads to the maintenance of homeostasis between the host and resident microbes.

During development and into adulthood, intestinal bacteria contribute to the shape and function of the gastrointestinal immune system [39] and play an important role in both health and disease [40]. This partnership involves bacterial signals that are recognized by host immune cells to mediate beneficial outcomes for both microbes and humans.

Another way to prevent the growth of pathogenic microorganisms is through the activation of the immune cells, such as macrophages, neutrophils, innate lymphoid cells 3 (ILC3), and B and T cells, to release antimicrobial factors. Commensal bacteria can also lead to SCFA production, enhancing the intestinal barrier function and stimulating mucus and antimicrobial peptides production [41]. In the same way, pathogenic bacteria also have mechanisms to prevent the growth of commensal bacteria. For example, some Gram-negative pathogenic bacteria have a secretion system dedicated to the protein secretion, such as type VI secretion system (T6SS) that is implicated directly in its pathogenicity and ability to kill their commensal competitors [42].

Stimulation of pattern-recognition receptors (PRR) present in intestinal epithelial cells (IEC), such as Toll-like receptor (TLR), NOD-like receptor (NLR), and RIG-like receptor (RLR), by commensal bacteria results in thymic stromal lymphopoietin (TSLP) production by these cells. TSLP can enhance B cell-activating factor (BAFF) and a proliferating-inducing ligand (APRIL) production. Additionally retinoic acid produced by dendritic cells (DCs) can promote IgA class-switching in B cells, and also is an important cofactor for the differentiation of Foxp3+ Tregs and has been shown to inhibit the generation of Th17 cells. IgA that is produced by lamina propria B cells is secreted into the intestinal lumen (SIgA), where it is able to alter microbiota composition and function [40, 41, 43].

Another important immune regulatory cytokine produced abundantly by IEC in the intestine is transforming growth factor-beta (TGF-*β*). IEC-derived TGF-*β* in combination with TSLP and retinoic acid promotes the conditioning of a subset of DCs found in the intestinal lamina propria and mesenteric lymph nodes that express the integrin *α* chain CD103 (CD103^+^ DCs) [44].

CD103^+^ express CCR7 that mediates homing to secondary lymphoid organs, drive the expression of gut-homing receptors CCR9 and *α*4*β*7 integrin on responding T cells, and induce differentiation of naive CD4+ T cells into FoxP3^+^ regulatory T cells [44, 45]. This subset of DCs is also the one that preferentially receives delivery of intestinal antigens by goblet cells at steady state which is consistent with their tolerogenic properties [46].

Interleukin-10 produced by DCs and macrophages also have the potential to induce Foxp3^+^ Tregs. The involvement of IL-10 in intestinal tolerance was confirmed in a model of experimental colitis. It has been shown that* B. fragilis* is able to prevent intestinal pathology by IL-10 production, and this cytokine is reduced within the gut-associated lymphoid tissue (GALT) of germ-free animals [47, 48]. A selected mixture of* Clostridia* species was shown to induce Tregs in the mouse colon, and oral administration of these species protected mice against colitis and allergic inflammation [49]. This indicates that commensal bacteria are involved in the promotion of FoxP3^+^ regulatory T-cell differentiation and maintaining intestinal tolerance [50].

Recently, it has been demonstrated that, in order to promote intestinal homeostasis, the commensal microbiota depends on the crosstalk between macrophages and retinoic acid receptor-related orphan receptor-*γ*t^+^ (RORyt^+^) ILC3. The microbiota stimulates macrophages to produce IL-1*β* that binds to the IL-1*β* receptor in ILC3s, promoting granulocyte-macrophage-colony stimulating factor (GM-CSF) release. ILC3-derived GM-CSF induces DCs and macrophages to produce regulatory molecules, such as IL-10 and retinoic acid [51].

In addition to its role in crosstalk with macrophages, ROR*γ*t^+^ ILC3 acts directly in the maintenance of the intestinal homeostasis and in the defense against intestinal pathogens. ROR*γ*t^+^ ILC3 are associated with IL-22 production, which can induce REGIII*γ* (C-type lectin antimicrobial peptides regenerating islet-derived protein) production by IECs. REGIII*γ* regulates the intestinal microbiota and contributes to the tolerance in the gut [52, 53]. At the same time, the commensal microbiota can induce IL-25 secretion by endothelial cells, which acts on lamina propria IL-17 receptor B (IL-17RB)^+^ DCs and suppresses IL-22 production by ROR*γ*t^+^ ILC3s [41]. It is a mechanism to ensure the maintenance of intestinal homeostasis.

Regarding adaptive immune response, the intestinal epithelium and underlying lamina propria contain T cells that play important role in maintaining intestinal homeostasis. T regulatory (Treg) cells are known to express the transcription factor forkhead box P3 (Foxp3) and suppress the activation, proliferation, and effector function of a wide range of immune cells, playing a key role in maintenance of intestinal homeostasis through anti-inflammatory cytokines such as IL-10 [54].

However, Treg cells are not homogeneous and terminally differentiated. A recent study demonstrated coexpression of ROR*γ*t and Foxp3 in Treg cells, which implies the conversion from Treg cells to Th17 cells, capable of producing IL-17. This is associated with a decreased suppressive function of Treg cells in patients with IBDs [55]. It was shown that Foxp3 is able to physically bind to ROR*γ*t and its transcriptional activity thereby blocking IL-17 production. But in the presence of appropriate inflammatory stimuli Treg cells display an IL17^+^ Foxp3^+^ CD4^+^ phenotype and can produce IL-17 [54].

However, when alterations in the normal microbiota, termed dysbiosis, occur in the gut, they lead to failure of the immune system regulation by commensal microbiota, resulting in an inflammatory state, with a predominance of Th1 and Th17 profile responses [41]. Inflammation in the intestine diminishs the tolerogenic characteristics of CD103^+^ DCs like the expression of the enzyme aldehyde dehydrogenase (ALDH) that participates in the conversion of retinal to RA and the expression of TGF-*β*. Conversion of Tregs is lower in this setting favoring a proinflammatory response with more production of the cytokine interferon-*γ* (IFN-*γ*) [56].

## 4. Resistance to Colonization by Commensal Microbes

As mentioned above, the microbiota is essential for modulating the immune system and some aspects of host metabolism. Therefore, changing the composition of the microbiota can be problematic for the host. Utilization of antibiotics as a treatment against bacterial infection has a huge impact in medicine [57–59]. Despite the benefits associated with antibiotic treatment, this therapy can change the microbiota for a long time. It has been reported that the combination regimen of amoxicillin, tetracycline, and metronidazole for two weeks induces an alteration in gut microbiota in patients with ulcerative colitis (UC) that lasts for three months [60]. In an experimental model, changes in the microbiota by metronidazole treatment were able to alter the integrity of the gut leading to exacerbation of* Citrobacter rodentium* infection [61]. In humans, hemorrhagic colitis can be associated with previous antibiotic treatment [62].

The fact that the presence of a normal microbiota inhibits the colonization of opportunistic pathogenic bacteria is called colonization resistance (CR) [63]. Colonization of the gut by pathogens such as* Salmonella typhimurium*,* Pseudomonas aeruginosa*,* Shigella flexneri*, and* Vibrio cholerae* is exacerbated by previous antibiotic treatment, showing the important role of the microbiota in inhibiting the attachment of these microorganisms to the intestine [64–66]. Interestingly, colonization of gnotobiotic mice with only one component of the microbiota is enough to control* Escherichia coli* colonization [67], and treatment with antibiotics can make conventional mice as susceptible as germ-free mice to colonization by* Salmonella* [68]. The mechanisms through which the microbiota can induce colonization resistance are not completely understood but may be associated with the systemic modulation of immune responses [69–71], and with the production of microbicidal substances [72–74]. Interestingly, the host immune response necessary to contain the pathogen could actually favor the growth of the pathogen and other harmful microbes by causing dysbiosis of the gut microbiome, and consequent impairment of colonization resistance mechanisms [75, 76].

## 5. Intestinal Dysbiosis

Breakdown of homeostasis in the gut environment causes dysregulation of intestinal immune responses and an imbalance of the normal intestinal bacteria called dysbiosis. The genetics of the host, as well as environmental perturbations such as antibiotic treatments, diet, or infections can influence the structure of the microbial community. These disturbances can lead to loss of diversity of the microbiota with a reduction in the commensals that are beneficial to the host and an increase in microbes that are potentially pathogenic. The importance of maintenance of diversity within the gut microbiota to gain maximum health benefits comes primarily from evidence that shows that members of the microbiota have diverse and nonredundant effects on host health. For example, the human symbiont* Bacteroides fragilis* directs the development of regulatory T cells and suppresses Th17 responses [77], whereas segmented filament bacteria (SFB) are able to induce production of IL-17 in the gut [47]. Thus, a dysbiotic gut microbiota represents a shift in the stability of the microbial community that is characterized by quantitative and qualitative changes in the composition, as well as in the local distribution of its members.

Recent studies have demonstrated an association between changes in the gut microbiota and acute mucosal infections, suggesting that they could act as a trigger for subsequent gastrointestinal disorders such as IBDs. Loss of diversity of the intestinal microbial community with increased abundance of Enterobacteria can be observed in several intestinal infections, such as* Citrobacter rodentium* [78],* Salmonella typhimurium* [76], and oral models of* Toxoplasma gondii*. Besides changes in the microbial composition, an exacerbated response to commensal signals is thought to be a major cause of pathology in experimental infections with* T. gondii* [79]. In* T. gondii *infection the changes in the microbiota aggravate the intestinal immune response caused by the parasite. In contrast, in* S. typhimurium *infection it seems that the alterations in the microbiota are not the cause but a consequence of the inflammatory process generated by the pathogen.

Acute infection with* T. gondii *causes translocation of bacteria from the intestinal lumen to peripheral tissues such as the spleen, mesenteric lymph node, and liver [80]. Disruption of intestinal homeostasis can lead intestinal bacteria to reach systemic sites in different settings. Microbial translocation, which is the translocation of microbial products from the gut lumen into the systemic circulation, and subsequent immune activation are thought to determine disease progression during HIV infection. Levels of plasma lipopolysaccharide (LPS), a marker of bacterial translocation, are increased in HIV infected patients [81]. Impairment of intestinal barrier integrity early in acute retroviral infection and loss of intestinal Th17 cells are probable causes of translocation in HIV infected individuals [82]. Furthermore, a shift in the gut commensal community was observed in HIV-infected subjects with overgrowth of Proteobacteria, which are known to have proinflammatory potential. The changes in the microbiota were associated with dysregulation of immune responses and consequent chronic inflammation [83]. In a humanized mouse model, treatment of irradiated recombination activating gene 2 (RAG2) deficient mice, which lack mature lymphocytes due to the inability to initiate V(D)J recombination, reconstituted with human cord blood cells with dextran sodium sulfate (DSS) induced bacterial translocation to the spleen and mesenteric lymph nodes [84].

Recently, an association of a genetic defect of the host and changes in the composition of the microbiota with nonalcoholic fatty liver disease steatohepatitis severity has been demonstrated revealing a role for inflammasomes in intestinal dysbiosis [85]. Inflammasomes are multiprotein complexes of innate immunity capable of recognizing a diverse range of conserved molecular motifs unique to microbes as well as tissue damage signals. Inflammasomes drive caspase-1 cascade activation which promotes secretion of proinflammatory cytokines IL-1*β* and IL-18 [86]. Alterations in the microbial profile were observed in the gut of mice deficient in the inflammasomes NOD-like receptor pyrin domain containing 6 (NLRP6) or NOD-like receptor pyrin domain containing 3 (NLRP3). Microbiota dysbiosis resulted in accumulation and recognition of bacterial products in the portal circulation through TLR signaling leading to hepatic steatosis and inflammation. In fact, the liver has been shown to have an important role in maintenance of compartmentalization of commensal intestinal microbes, clearing bacteria that reach systemic vascular circuits. In both animal models and human patients with liver disorders, loss of this function leads to aberrant immune responses against gut commensals [87].

More recently, profiling studies of the microbiota have associated pathogenicity of inflammatory diseases with distinct shifts in the composition of the intestinal microbiota. Assessment of intestinal commensals in type II diabetes patients revealed a moderate degree of dysbiosis with a decrease in butyrate-producing bacteria and an increase in several opportunistic pathogens [88]. Studying the microbiome of a large pediatric cohort of Crohn's disease (CD) patients prior to treatment, Gevers and colleagues observed increased abundance of Enterobacteria and amplification of dysbiosis after antibiotic use [89]. These authors suggested that screening of the microbiota profile at an early stage of the disease could be a useful diagnostic tool for CD. Since diagnosis of IBD is particularly challenging in children due to variations in symptoms, enhanced technologies that could rapidly identify microbial patterns associated with development of the disease would be very important [90].

A common hallmark of intestinal microbiota dysbiosis is the outgrowth of opportunistic pathogens or also called pathobionts. This phenomenon could be explained by recent evidence that suggests that inflammation in the intestine establishes a nutritional local environment that is better suited for the growth of certain microorganisms. It is probable that these potentially pathogenic microbes are more capable of utilizing the nutrients that are generated by the inflammatory process [91]. Furthermore, bacteria might adapt to growth in dysbiotic conditions and acquire even higher pathogenic potential by horizontal gene transfer of virulence factors, indicating that disruption of the intestinal homeostasis and consequent changes in the microbial community could contribute to pathogen evolution [92]. Thus, preventing dysbiosis, especially in the hospital environment, may have an even more fundamental role for the control of emerging infectious diseases.

The homeostatic relationship between host and microbiota does not imply that microorganisms are not continually sensed by the host immune system. Recognition of small numbers of commensal bacteria and their products that are probably continuously penetrating the intestinal epithelial cell layer and may result in protective adaptive immune responses being induced in the intestinal mucosa [93]. In fact, the stimulatory capacity of the microbiota has been shown to be important in maintaining responsiveness against pathogenic microbes [70, 94].

Disruption of intestinal homeostasis by intestinal inflammatory disorders such as IBDs or gastrointestinal infections has been previously linked with newly acquired responsiveness against antigens from normal gut bacteria. In fact, it has long been reported by several groups that the systemic adaptive immune system can indeed be primed against gut bacterial antigens [95–97]. Interestingly, commensal-specific responses are observed in healthy individuals, suggesting that commensal recognition is a common occurrence and, in most circumstances, is not associated with pathogenic responses [98]. Therefore, tolerance towards commensals is maintained in a healthy gut. Whether microbiota-specific responses could be detrimental in the context of dysregulation of the intestinal homeostasis is not known. Recent data suggest that acute infections may result in the disruption of tolerance to gut microbes. Experimental* T. gondii* ileitis leads to translocation of bacteria and generation of T cells specifically against commensal antigens. These cells are long-lasting and capable of proliferating and become activated upon antigen recognition [80]. Despite the clear association between commensal-specific responses and inflammatory disorders, whether acute mucosal infections could function as a trigger for the development of IBDs remains to be addressed. Gaining further insight of how recognition of bacteria in the gut influences immune responses could help understand how intestinal inflammatory disorders occur and may also permit the development of new strategies to prevent the onset of such syndromes.

## 6. The Role of the Intestinal Microbiota in Inflammatory Bowel Disease

Inflammatory bowel disease is an immune-mediated disorder that is characterized by chronic intestinal inflammation and which encompasses primarily ulcerative colitis and Crohn's disease (CD). Bloody, mucous diarrhea is the almost universal hallmark of UC [99]. Symptoms of CD are more subtle and varied, partly as a result of its diffuse and diverse anatomical location. The most common symptom is abdominal pain [100]. However, there are other associated symptoms, such as diarrhea, poor appetite, and weight loss. These symptoms are presented in nearly 80% of children and adolescents with IBDs.

Etiologic factors have been associated with different environmental aspects that contribute to inflammatory bowel diseases such as smoking and appendectomy. Vitamin D levels, diet, hormone use, and stress have also been postulated as risk factors for one or both main forms of IBDs, but these factors need to be further investigated [99, 101].

The critical function of adult gut performance is related to the metabolism of dietary components, such as cholesterol, intestinal motility, and immune system modulation [101, 102]. Preserving eubiosis, which is the state of equilibrium of the microbiota in the gastrointestinal tract, is relevant for maintaining the integrity of the intestinal epithelium and contributing to antimicrobial defenses [101]. Microbe-induced Treg cells that prevent potential inflammatory responses by both adaptive and innate immunity responses also promote homeostasis. Some problems in homeostasis may result in an anomalous activation of some innate receptors and subsequent tissue damage, leading to systemic inflammation that results in symptoms associated with IBDs. For example, IBD is related to a dysfunctional immune response and activates T-helper cells in the gut mucosa, probably because of the deregulation of the normally controlled immune response to commensal bacteria. It is important to note that the number of commensal bacteria is reduced in patients with IBD [102].

Several studies have shown protection of the gut against external bacteria by commensal microbes, supporting their function in the etiology of IBDs [101]. For example, CD was associated with a reduction in the antibacterial peptide expression. These factors can explain the association between maintenance of inflammatory responses to intestinal pathogens and loss of tolerance to commensal microbiota [101].

The NOD2 signaling pathway is presented and is important as a regulatory factor of proinflammatory proteins induced by NF-*κ*B. After proinflammatory stimuli such as TNF-*α* and IFN-*γ*, the expression of NOD2 may be upregulated in epithelial cells, including those of the gastrointestinal tract. It has been postulated that the decrease in the function of NOD2 reduces the responsiveness of the host to pathogens, culminating in chronic intestinal inflammation. The impaired function of this receptor facilitates the invasion of bacteria and changes the mucosal immune responses against gut luminal antigens [103]. Taking the example of Crohn's disease (CD), genetic studies have begun to elucidate the loci associated with subphenotypes of the disease, as the location of the disease and clinical outcome. It has been suggested that patients with CD have mutations in NOD2 and thus poorly respond to bacterial antigens [104].

## 7. The Role of Gut Commensals in Colorectal Cancer

Several cancer types are associated with infectious agents. Well-known examples include cervical and gastric cancer, which can be caused by human papillomaviruses and the bacteria* Helicobacter pylori*, respectively [105, 106]. It is becoming increasingly evident that the gut bacterial population plays an important role in colon carcinogenesis [17].

Studies of fecal microbiota of 19 patients with colorectal cancer (CRC) and 20 healthy control subjects demonstrated differences in the fecal microbial composition between these two groups. The CRC group had a significant increase in the relative abundance of Fusobacteria phyla compared with the control group. Regarding Bacteroidetes and Firmicutes phyla, no difference was observed in their relative abundance. However, a positive correlation between the abundance of* Bacteroides* species and CRC was observed [107].

Other studies have also demonstrated that the genus* Bacteroides* had higher rates of colonization in CRC patients [107, 108]. A possible mechanism could be through the release of enterotoxins, such as fragilysin, an oncogenic bacterial toxin [109]. Fragilysin-producing* B. fragilis*, termed enterotoxigenic* B. fragilis* (ETBF), found in colonic biopsy specimens has been demonstrated to have a significant correlation with the presence of active inflammatory bowel disease [110, 111]. Fragilysin is able to induce a gut inflammatory state. Fragilysin can stimulate IL-8 secretion by intestinal epithelial cells and stimulates expression of the neutrophil chemoattractant and activators epithelial cell-derived neutrophil attractant 78 (ENA-78) and growth related oncogene *α* (GRO-*α*) [112–114]. In addition to its inflammatory effects, fragilysin induces colonic epithelial cell proliferation, as well as expression of the oncogene c-Myc [115].

Gut microbial profiling of germ-free IL-10-deficient mice that develop spontaneous colitis revealed that intestinal inflammation induces changes in the composition of the microbiota with an overgrowth of Enterobacteria. Monoassociation with the commensal murine adherent-invasive* E. coli* NC101 contributed to the development of invasive tumors in germ-free IL-10-deficient mice treated with the colon-specific carcinogen azoxymethane (AOM). Deletion of the virulence factor polyketide synthase (Pks) genotoxic island of* E. coli* NC101 reduced numbers of tumors and invasion in mice, and presence of Pks^+^ *E. coli* NC101 was associated with patients with IBD and CRC, suggesting that colitis-induced dysbiosis and expansion of virulence microbes can lead to tumorigenesis [116].

## 8. Intestinal Infections and the Microbiota

The gut flora usually contributes to a healthy environment. However, pathogenic and commensal bacteria are responsible for acute and chronic inflammation of the mucosa, influencing both the innate and adaptive immune responses [117].

### 8.1. *Salmonella typhimurium*

Members of the* Salmonella* genus are a diverse group of facultative intracellular gram-negative organisms that are responsible for a broad spectrum of enteric and systemic diseases found in humans and other vertebrates.* S. typhimurium* is a common pathogen found in humans and causes acute gastroenteritis [118]. Also,* Salmonella* causes invasive infections, such as enteric fever, septicemia, and osteomyelitis. The virulence of these bacteria depends on their serotypes, the state of the host, and the size of inoculum. Additionally,* Salmonella* has the ability to change the process of phagocytosis [119, 120]. Upon entry into the human host,* Salmonella* spp. must overcome the resistance to colonization mediated by the gut microbiota and the innate immune system. These bacteria successfully accomplish this by inducing inflammation and mechanisms of the innate immune defense. Many models have been developed to study* Salmonella* spp. interactions with the microbiota and these have helped to identify factors necessary to overcome colonization resistance and to mediate disease. Microbiota-produced butyrate and acetate can have dramatic effects on both the host and* Salmonella* spp. during infection [121].

*Salmonella typhimurium* has been shown to be unable to colonize the mouse intestine in the absence of inflammation, as the normal microbiota in the noninflamed state is able to effectively outcompete an avirulent (lacking inflammatory capacity)* Salmonella* intruder [76, 91]. Other studies have found that different antibiotics have variable effects on the total number and distribution of gut bacteria but that each antibiotic tested enhanced* Salmonella*-induced epithelial cell invasion and inflammation [122]. After antibiotic removal and some recovery of the microbiota, mice were still susceptible to* Salmonella*-induced enteritis, suggesting that the correct balance of microbial diversity and numbers is required for effective colonization resistance.

### 8.2. Pathogenic* Escherichia coli* and* Citrobacter rodentium*

Enteropathogenic* E. coli* (EHEC) and enterohemorrhagic* E. coli* (EPEC) are human diarrheal pathogens that cause much morbidity and mortality worldwide. Unlike the harmless commensal strains of* E. coli* that reside in the human intestine, pathogenic strains of* E. coli* are highly adapted enteric bacteria that have specific virulence determinants such as a pathogenicity island called the locus of enterocyte effacement (LEE) which leads to the formation of attaching and effacing (A/E) lesions. EHEC strains also are able to produce several cytotoxins [123]. EHEC causes inflammation in the large intestine, whereas EPEC affects mainly the proximal small intestine.* Citrobacter rodentium* is a natural pathogen found in mice that carries a homolog of the LEE pathogenicity island of EPEC and EHEC and, therefore, is used as a model to study the molecular basis of pathogenic* E. coli* infections. Unlike the harmless commensal* E. coli* that reside in the human intestine, pathogenic* E. coli* are highly adapted enteric bacteria that have evolved to use attaching and effacing (A/E) lesion formation as a major mechanism of infection [124].

Although the commensal microbiota has crucial roles in resistance to enteric pathogen infections, certain pathogens can use the microbiota to facilitate their infection. Commensals may have a direct role in controlling pathogenic bacteria. For example,* Bifidobacterium* species directly inhibit the growth of EHEC by acidification of the local environment [125]. Commensal* E. coli* can compete for nutrients against EHEC strains [126]. The microbiota is also involved in the ability of* C. rodentium* to colonize the intestine, since germ-free mice are unable to clear the bacteria. During the late phase of the infection, virulence factors of* C. rodentium* are downregulated and the bacteria are outcompeted by the microbiota [127]. Additionally, recent findings suggest that the microbiota is important for* C. rodentium* resistance mediated by the production of IL-22 [128].

### 8.3. *Clostridium difficile*

*Clostridium difficile* is an opportunistic pathogen of humans that causes intestinal infections named CDI (*Clostridium difficile* infection). This infection is a major cause of diarrhea and antibiotic-induced colitis. There are classical manifestations associated with CDI, such as the progression of mild diarrhea to fulminant colitis and toxic megacolon. Infections caused by this microorganism are correlated with the decrease of commensal organisms in the gut [129]. Antibiotics are also linked with this pathogen, and an inappropriate and excessive use of antibiotics predisposes toward development of the infection [130].

Patients over 65 years hospitalized with recent antibiotic exposure present the highest risk of developing this infection. Studies showed that reduction of Bacteroides and Firmicutes phyla in the gut caused by antibiotics seems to be important in understanding* C. difficile* pathophysiology [119, 131].

One of the strategies to treat CDI, especially in recurrent cases, is fecal microbiota transplantation. This technique is based on the transplantation of a microbiota obtained from a healthy donor. The sample is processed and transplanted into patients with recurrent CDI. This is a successful technique that provides a >90% success rate. An example of its effectiveness is that symptoms of infection caused by* C. difficile* are mostly resolved after the procedure [129].

## 9. Effects of Probiotics

Probiotics are “live microorganisms which, when administered in adequate amounts, confer health benefits to the host” [132]. Therefore, to fulfill their objectives, these microorganisms should resist the adversities of the host organism, stomach pH, and bile salts, until they reach the intestine. Beneficial effects of these microorganisms and their safety to the host must be proved. In addition, they should be stable and viable from the start of production to consumption. The major microorganisms currently utilized as probiotics are bacteria of the genera* Lactobacillus* and* Bifidobacterium* and the yeast* Saccharomyces boulardii*. Probiotics are currently being consumed in supplemented foods, fermented milks, and yogurts [133–135], and also ingested with medicines, as discussed by Vieira and collaborators [136].

When they reach the gut, probiotics can act in several ways. One of them is in the intestinal lumen by stimulating mucin production, defensins, and bacteriocins [137, 138]. Other mechanisms of action include the ability to maintain and modulate intestinal homeostasis by enabling survival of cells during intestinal infections by pathogens, preventing bacterial translocation, competing with pathogens for space and nutrients, reducing intestinal permeability, and producing or inducing the production of lactate and acetate. In addition, they can affect the metabolism of the microbiota [125, 134, 139, 140]. Modulation of host immunity is another benefit of probiotics consumption. Probiotic microorganisms are able to stimulate the immune system, either the innate immune responses, by inhibiting signaling pathways, such as the MAPKs [138, 141] and NF-kB [94, 142] and by altering the profile of secreted cytokines [143, 144], or the adaptive immune responses, by stimulating T lymphocytes [145, 146].

Studies in animal models and human clinical studies have generated a positive outlook for the use of probiotics in the prevention and treatment of several diseases. The use of probiotics in murine models of IBD and clinical studies of this disease has not shown significant results, except for an improvement of symptoms in some cases [147, 148]. In murine cancer models, probiotics promoted inactivation of mutagenic compounds suppressing pre-cancerous lesions [149], inhibition of development of cancer cells [145, 150], and a reduction in the size and number of tumors [151]. Moreover, the use of probiotics in a human study showed a reduced risk of developing colorectal cancer [152].* Saccharomyces boulardii* promoted a reduction in the duration of diarrhea in children without specific etiology [153], and administration of* Lactobacillus rhamnosus* GG reduced the duration of diarrhea caused by rotavirus in children [154]. Another study showed positive results for antibiotic-associated diarrhea when* Bifidobacterium lactis* and* Streptococcus thermophilus* were administered to children [155].

There are no reports in the literature of negative effects of probiotics in healthy people. All negative effects have been observed in critically ill, hospitalized or postoperative patients. Immunosuppression and prior antibiotic treatment were shown to be predisposition factors in cases of* Lactobacillus* bacteremia. Importantly, the consumption of* Lactobacillus* did not increase the incidence of bacteremia during a 10-year study [156]. Patients admitted to the intensive care unit (ICU) developed fungemia following use of* Saccharomyces boulardii *[157] and the same result was observed in neutropenic patients [158]. Children with short bowel syndrome developed sepsis associated with use of probiotic* Lactobacillus rhamnosus* GG [159], and acidosis due to the production of D-lactate during bacterial fermentation [160].* Lactobacillus rhamnosus* GG induced sepsis in a patient who underwent a cardiac surgery [161]. Probiotics constitute a source of antibiotic resistance genes.* In vivo *transfer of these genes to bacteria in the gastrointestinal tract has been reported in mice and humans [162]. Evaluation of the transferability of resistance genes is important to determine the full safety of a probiotic strain.

Understanding the molecular mechanisms through which probiotics act in the gut, altering the host physiology and modulating the immune system, could lead to the development of more successful therapies for various disorders. Furthermore, it is important to characterize which microorganism presents the best results for a particular disease. Research with microorganisms is progressing, and the clinical safety and efficacy of the use of probiotics need to be confirmed.

## 10. Conclusion

The gastrointestinal tract is the primary site of interaction between the host immune system and commensal and pathogenic microbes. A large body of evidence has now been gathered confirming the fundamental role of gut commensal microbes in the maintenance of intestinal homeostasis. The gut microbiota is a complex community of symbiotic microorganisms that is highly susceptible to disturbances. Dysregulation of intestinal homeostasis leads to loss of microbial diversity, overgrowth of pathobionts, and translocation of bacteria. Commensal dysbiosis and consequent abnormal sensing of commensal bacterial antigens is associated with the pathogenesis of various disorders. Although both genetic and environmental factors are involved, the molecular mechanisms responsible for triggering dysbiosis are still largely unknown. Furthermore, whether these changes are specific to each disease needs to be addressed. Probiotics have been successfully used as a strategy to regulate an altered microbiota and provide important signals to activate proper immune responses in several inflammatory disorders, gastrointestinal infections and cancer. A better understanding of how disturbances in the intestine can affect intestinal homeostasis resulting in atypical responsiveness against commensal bacteria could provide new important insights into the etiology of inflammatory diseases, such as IBD, and may contribute to the development of new strategies for prevention and therapy of these disorders.
